# Screening Tools and Assessment Methods of Cognitive Decline Associated With Age-Related Hearing Loss: A Review

**DOI:** 10.3389/fnagi.2021.677090

**Published:** 2021-07-14

**Authors:** Tao Yue, Yu Chen, Qi Zheng, Zihao Xu, Wei Wang, Guangjian Ni

**Affiliations:** ^1^Department of Biomedical Engineering, College of Precision Instruments and Optoelectronics Engineering, Tianjin University, Tianjin, China; ^2^Tianjin International Engineering Institute, Tianjin University, Tianjin, China; ^3^Academy of Medical Engineering and Translational Medicine, Tianjin University, Tianjin, China; ^4^Department of Otorhinolaryngology Head and Neck Surgery, Tianjin First Central Hospital, Tianjin, China

**Keywords:** age-related hearing loss, cognitive decline, presbyacusis, EEG, MRI

## Abstract

Strong links between hearing and cognitive function have been confirmed by a growing number of cross-sectional and longitudinal studies. Seniors with age-related hearing loss (ARHL) have a significantly higher cognitive impairment incidence than those with normal hearing. The correlation mechanism between ARHL and cognitive decline is not fully elucidated to date. However, auditory intervention for patients with ARHL may reduce the risk of cognitive decline, as early cognitive screening may improve related treatment strategies. Currently, clinical audiology examinations rarely include cognitive screening tests, partly due to the lack of objective quantitative indicators with high sensitivity and specificity. Questionnaires are currently widely used as a cognitive screening tool, but the subject’s performance may be negatively affected by hearing loss. Numerous electroencephalogram (EEG) and magnetic resonance imaging (MRI) studies analyzed brain structure and function changes in patients with ARHL. These objective electrophysiological tools can be employed to reveal the association mechanism between auditory and cognitive functions, which may also find biological markers to be more extensively applied in assessing the progression towards cognitive decline and observing the effects of rehabilitation training for patients with ARHL. In this study, we reviewed clinical manifestations, pathological changes, and causes of ARHL and discussed their cognitive function effects. Specifically, we focused on current cognitive screening tools and assessment methods and analyzed their limitations and potential integration.

## Introduction

According to the statistics of the World Health Organization, almost one-third of all adults above 65 years of age are affected by hearing loss, with 226 million experiencing disabling hearing loss. With the rise and aging of the global population, the number of people with hearing loss is snowballing (World Health Organization, 2018), and as per current estimates, this number is expected to rise to almost 585 million by 2050. Hearing loss in the elderly mostly involves age-related hearing loss (ARHL), which refers to the sensorineural hearing loss occurring with age (Slade et al., 2020). Patients with ARHL have difficulty processing voice information and perceiving speech, which causes communication barriers and sensory deprivation. Some individuals with ARHL will avoid social interaction, which aggravates loneliness and depression, leading to social isolation. Researchers found that ARHL is associated with cognitive decline. However, the exact correlation mechanism between hearing loss and cognitive decline has not yet been fully elucidated to date. In this review, we summarized the hypotheses regarding the relation between ARHL and cognitive decline and discussed physiological and clinical manifestations of ARHL.

### Pathology and Characteristics of Age-Related Hearing Loss

According to the results of pure tone audiometry and changes in temporal bone histology, ARHL is divided into the following types (Schuknecht and Igarashi, 1964): (1) Peripheral ARHL, where the main changes are the loss of outer hair cell at the base of the cochlea, spiral ganglion cell, and auditory nerve fiber (Ohlemiller, 2004; Wu et al., 2019). Typical clinical manifestations are reduced speech recognition ability and a steep drop in high-frequency hearing. (2) Metabolic or Vascular ARHL, where the main pathological changes include atrophy of the spiral ligament and stria vascularis (Wiwatpanit et al., 2018). Typical clinical manifestations are progressive hearing loss, whereas speech recognition ability is not significantly reduced. (3) Mechanical ARHL, where the main pathological changes include basilar membrane sclerosis and degeneration of cochlear nerve fibers (Keithley, 2020). This is characterized by a high-frequency hearing loss that is severe but does not affect daily communication. (4) Central ARHL, involving mainly degenerative changes in the central nervous system function, where clinical manifestations are a distortion of the sound perception of the surrounding environment and obstacles to sound localization. (5) Mixed ARHL, for most patients with ARHL, their clinical manifestations are often mixed, and there is more than one histological change.

**Figure 1:**
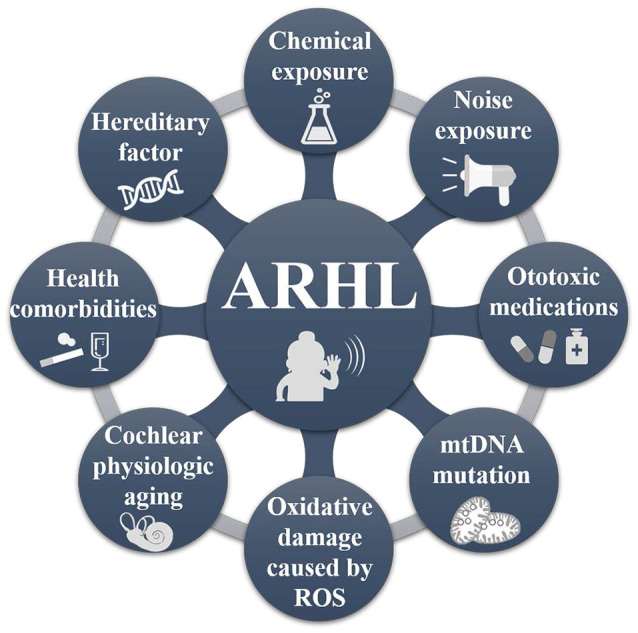
The pathogenic factors of age-related hearing loss (ARHL). ARHL is a multifactorial disease, mainly caused by external environmental factors (e.g., noise and exposure to chemical factors, ingestion of ototoxic medications, intake of hormones, alcohol, nicotine, etc.); mitochondrial DNA deletion mutation (Seidman, 2000; Yamasoba et al., 2013; Lyu et al., 2020); metabolic factors (oxidative damage caused by reactive oxygen species, related cell apoptosis; Pickles, 2004; Fetoni et al., 2018); physiological aging of the cochlea and genetic factors (Liu and Yan, 2007; Ciorba et al., 2015; Tawfik et al., 2020). The mutual influence of these factors leads to the cumulative development of ARHL.

The pathogenesis of ARHL is highly complex, as studies have shown that it may result from multi-link and multi-factor interaction, involving various aspects of human physiology, pathology, biochemistry, and molecular biology (Figure 1; Rousset et al., 2020). For most patients with ARHL, among the most evident symptoms is a decline in speech recognition ability. Patients are able to hear surrounding sounds but have difficulty distinguishing and understanding them. Over time, patients with ARHL enter into a state of auditory deprivation, and daily communication between patients and people becomes increasingly difficult, which manifests as a decline in social adaptability, a change in mental state, sense of social isolation, and decline in the quality of life (Gates and Mills, 2005; Huang and Tang, 2010).

### Cognitive Decline Associated With ARHL

The impact of ARHL on the elders, however, may not only be reflected in their hearing ability. As one of the clinical manifestations of ARHL described above, the patients’ executive function and psychomotor processing change as well (Quaranta et al., 2014). Hearing impairment makes daily communication between patients and others more difficult. This profoundly affects interpersonal communication, independence, happiness, and quality of life (Anon, 2016). It reduces the patients’ social adaptability, causes changes in the mental state (Bowl and Dawson, 2019), and may lead to social isolation, depression, and possible cognitive impairment (Fortunato et al., 2016; Cosh et al., 2019; Jafari et al., 2019). A meta-analysis of 11 cohort studies found that suffering from peripheral or central hearing impairment was associated with a higher risk of cognitive impairment, and the risk increased with the degree of hearing loss: for moderate/severe hearing impairment, the risk increases by 1.57 times (95% confidence interval, CI: 1.13–2.20); for severe central hearing impairment, the risk increases by 3.21 times (95% CI: 1.19–8.69; Yuan et al., 2018). According to the Lancet Commission 2020 report, if hearing impairment, which is one of 12 modifiable risk factors for dementia, is eliminated, the risk of dementia is reduced by 8%. The report claims that the risk of dementia increases by 30% (95% CI: 1.00–1.60) per 10 dB of worsening of hearing loss (Livingston et al., 2020). A meta-analysis of 40 studies reported that ARHL was a possible biomarker and modifiable risk factor for cognitive decline, cognitive impairment, and dementia (Loughrey et al., 2018). A 13-year longitudinal study of 9,666 adults above the age of 50 from the United Kingdom evaluated cognitive function every 2 years and found that the direct association between mild hearing loss and memory test was –0.52 (95% CI: –0.65 to –0.39) (Ray et al., 2018). Moreover, a study reported that it is possible to prevent cognitive decline associated with ARHL through early auditory intervention and increased opportunistic screening for the elders (Davis and Smith, 2013). Cumulative evidence strongly suggests that ARHL independently increases the risk of cognitive impairment and dementia, increasing with the severity of hearing loss (Panza et al., 2019).

Although a link between hearing loss and cognitive decline has been acknowledged, and numerous studies have been conducted on the possible correlation between ARHL and cognitive impairment, the relationship between the two remains unclear. Presently, there are three main hypotheses, namely the: (1) common cause hypothesis; (2) cascade hypothesis, and (3) cognitive load hypothesis (Lin and Albert, 2014). Researchers supporting the common cause hypothesis argue that there is a third variable that causes the seniors’ multiple sensory and cognitive declines at the same time, i.e., that hearing impairment and cognitive decline have common age-related changes, such as the central nervous system degeneration (Wayne and Johnsrude, 2015). The cascading hypothesis suggests that prolonged continuous hearing loss leads to social isolation, loneliness, apathy, and depression, which cause a reduction in cognitive stimulation and thus impaired cognitive function. Imaging studies of some patients with hearing loss showed that after hearing loss was aggravated, the auditory cortex atrophied, and the brain volume decreased to a certain extent, which might also reduce the brain’s ability to perform other tasks besides hearing (Golub, 2017). All of these factors further accelerate the decline of cognitive function. The cognitive load hypothesis argues that hearing loss will lead to decreased quality of auditory signals received by patients, and further cognitive resources will be consumed in the process of auditory perception. In this manner, the higher listening effort will decrease the performance of patients in other cognitive tasks during the listening process (Chern and Golub, 2019). In the long term, high-load tasks may also eventually lead to the depletion of cognitive reserves, manifesting as cognitive decline.

### Role of Assisted Listening Device

Because ARHL is an irreversible degenerative disease, there is currently no effective treatment. In particular, patients with moderate-to-severe hearing loss, who have a long onset time, often rely on assisted listening devices, such as hearing aids and cochlear implants, to improve their hearing level (Sprinzl and Riechelmann, 2010; Löhler et al., 2019). Hearing aids are suitable for patients with mild-severe hearing loss and are the first choice for patients with ARHL (Williger and Lang, 2014; Ferguson et al., 2017). The working principle of hearing aids is to amplify the external sound to the degree required by the hearing loss patient and use the patient’s residual hearing to obtain greater stimulation to compensate for the hearing loss (Dillon, 2008). Cochlear implants work by converting external acoustic signals into electrical signals and directly stimulate auditory nerve fibers through electrodes implanted in the cochlea, thereby restoring the patient’s auditory function (Roche and Hansen, 2015). Cochlear implants are generally suitable for patients with severe-to-profound hearing loss and are significantly more effective than hearing aids in language understanding (Jiam et al., 2017; Rapport et al., 2020; Svirsky et al., 2020). However, because most patients are elderly, they may be concerned about whether the potential complications of cochlear implant surgery outweigh the benefits. A 13-year retrospective comparative study of the clinical and functional effects of cochlear implantation in the elderly found that patients’ overall quality of life after cochlear implantation was significantly improved (*p* < 0.001), such that the patients’ age should not be a factor in deciding whether to receive a cochlear implant (Orabi et al., 2006). A research report on the outcomes after cochlear implantation in the very elderly likewise showed that speech perception benefited from cochlear implants, and age was not a limitation for the implant (Wong et al., 2016).

Considering this association between hearing loss and cognitive decline, researchers studied whether an assisted listening device can ameliorate the currently observed risk of accelerated cognitive decline due to hearing loss in older adults. A longitudinal cohort study designed to test whether the use of hearing aids can change the cognitive trajectory of the elderly, which tested the cognitive performance of 2,040 people above 50 every 2 years for 18 years, found that the patients’ decline in episodic memory decelerated after using a hearing aid (*β* = –0.02, *p* < 0.001; Maharani et al., 2018). Long–term follow-up studies demonstrated that hearing aids might have a mitigating effect on the trajectory of cognitive decline in later life. A large cross-sectional study of 164,770 adults also found that hearing aid usage was associated with better cognitive performance (Dawes et al., 2015b). These research results lean toward the cascade and cognitive load hypotheses. This because if the cognitive function is assumed to be affected by the deterioration of the central nervous system based on the common cause hypothesis, no matter whether maintaining a fair hearing or using assisted listening devices, it will not affect the rate of cognitive decline. These results are encouraging, indicating that using assisted listening devices positively affects cognitive function and can help reduce the risk of cognitive decline in patients with ARHL. However, the cognitive benefit of hearing aids could also be due to a recruitment bias, as elders with better cognitive function are more prone to use hearing aids (Glick and Sharma, 2020; Vogelzang et al., 2021).

Furthermore, not all results are in agreement. A follow-up study of 16 elders with cochlear implants argued there was no significant cognitive function change (Sonnet et al., 2017). Although this study’s maximum follow-up time was 12 months, extensive further research is urgently required to determine the benefits of treating hearing loss on cognitive outcomes.

Certainly, assisted listening devices can improve the life quality of hearing-impaired people, so they are still strongly recommended to treat hearing loss. Although the prevalence of hearing loss is very high, the usage rate of hearing aids remains low. A statistical survey among 1,503 participants who required hearing aids estimated a prevalence of hearing aid acquisition at only 6.5% (95% CI, 5.3–7.8; He et al., 2018). According to the report, the main reason for individuals not acquiring a hearing aid was the feeling that it was unnecessary, incomprehension, and unaffordability. Given the benefits of hearing aids to hearing, quality of life, and the potential benefits of cognitive function, most of all, the use of hearing aids is an accessible strategy. It is necessary to improve and disseminate knowledge on hearing and enhance understanding of hearing aid function among hearing loss patients.

## Cognitive Screening Tools Overview

### Questionnaire

A variety of cognitive screening tools have been used to study the relationship between ARHL and cognitive decline (Shen et al., 2016). The most widely used and studied cognitive function screening tool is the mini-mental state examination (MMSE; Folstein et al., 1975), which includes 30 questions investigating the aspects of orientation, registration, attention, and calculation. A score greater than or equal to 24 (total score 30) denotes normal intelligence. The standard score can be modified for years of education and age. The questionnaire has the advantages of being short, easy to manage and score, and is often used as a reference for comparative evaluations of other assessments. Another commonly used questionnaire is the Montreal cognitive assessment (MoCA; Nasreddine et al., 2005), which contains 11 examination items in eight cognitive fields, including attention, concentration, executive function, memory, language, visuospatial abilities, abstraction, calculation, and orientation (Lim and Loo, 2018). A score greater than or equal to 26 (total score 30) indicates normal intelligence. This questionnaire is more sensitive than MMSE in detecting mild cognitive impairment (Saczynski et al., 2015; Ciesielska et al., 2016), and the test time is shorter, which is more suitable for clinical application.

However, these questionnaires’ performance may be negatively affected by hearing loss, leading to false-positive recognition of cognitive impairment, thereby overestimating the degree of cognitive decline (De Silva et al., 2008; Dupuis et al., 2015). A study developed a modified questionnaire (HI-MoCA) designed explicitly for hearing loss patients based on MoCA to convert verbal instructions into visual ones (Lin et al., 2017). However, this is more complicated than the original MoCA, and it is not clear whether such changes would affect the specificity and sensitivity of the detection. Further research is still encouraged to use appropriate cognitive screening tools for hearing loss patients and perform appropriate statistical tests.

Notably, the cognitive assessment of ARHL patients using questionnaires has an evident drawback, namely, the results are subjective (Jayakody et al., 2018). Because the final evaluation result may be influenced by the patient’s understanding of the problem and the environment, an objective quantitative index would be more capable of reflecting the patient’s cognitive status truly.

### Electrophysiologic Method

ARHL and cognitive impairment are complex multifactorial diseases, and the two may influence each other during the research process. Hearing loss will affect the cognitive test scores and can thus be mistaken for cognitive impairment, while cognitive impairment may also affect the auditory function test results, thereby exaggerating the degree of hearing loss. Hence, it is promising to employ objective electrophysiological tools to assess patients diagnosed with ARHL. Clinically, common electrophysiological tools for patients with ARHL include the electrocochleogram (ECochG), auditory steady-state response (ASSR), otoacoustic emission (OAE), and auditory brainstem responses (ABR). Currently, these are powerful tools to objectively assess the degree of hearing loss, cochlear function, and auditory nerve–auditory pathway in patients. A limited number of studies have correlated OAE (Belkhiria et al., 2019, 2020) and ABR (Delano et al., 2020) with magnetic resonance imaging (MRI) which is the most commonly used objective measurement tool of cognitive function. Furthermore, electroencephalogram (EEG) technology has become another potential tool to measure cognitive function. Here, we focus on the advantages, prospects, and drawbacks of MRI and EEG in this field.

#### Magnetic Resonance Imaging

MRI, particularly structural MRI (Hamilton et al., 2019), and functional MRI (fMRI), are powerful tools for studying the neural mechanisms of disease (Yousaf et al., 2018). An MRI and diffusion tensor imaging study involving patients with ARHL and age-matched normal-hearing participants found that the gray matter volume in the frontal cortex was significantly lower in the ARHL group than in the control group (Rosemann and Thiel, 2020). The results suggested that the cortical gray matter atrophy observed in the brains of older people with hearing loss is independent of age. A 6-year longitudinal study of brain volume detection of the elderly with hearing loss found that compared with a normal-hearing control group, the elderly with hearing loss showed an accelerated decline in the overall brain volume, particularly in the right temporal lobe (Lin et al., 2014), which suggested that differences in the cortical structure are related to the duration of hearing loss.

In addition to MRI studies on the local brain regions of patients with ARHL, the whole-brain functional network’s research deserves further discussion. An MRI study on the functional connectivity of the resting state of 65 patients with ARHL found that a higher degree of hearing loss was significantly associated with decreased resting-state functional coupling, and the connectivity of the dorsal attention network gradually decreased with an increase in the hearing loss degree (Schulte et al., 2020). The connectivity of the dorsal attention network increases when performing tasks requiring attention and responding to stimuli (Corbetta and Shulman, 2002). Impaired connectivity in the dorsal attention network may explain the risk of cognitive decline in patients with ARHL.

In summary, MRI studies confirmed that ARHL significantly alters the cochlear function in the peripheral auditory system and profoundly affects neural processing in the brain (Peelle and Wingfield, 2016; Belkhiria et al., 2019). The volume of the overall brain and the local volume of the right temporal lobe of ARHL patients decreased at an accelerated pace, the cortical volume of the auditory processing area of the superior temporal gyrus was reduced, and the connectivity of the dorsal attention network decreased gradually (Qian et al., 2017; Rosemann and Thiel, 2019). These areas are crucial for verbal processing, semantic memory, concentration, and sensory integration. Changes in the brain structure observed in the elderly with hearing loss cannot be fully explained by age-related mechanisms, providing evidence for the cascade and cognitive load hypotheses.

#### Electroencephalogram

In 1929, Berger (1929), a German psychiatrist, first used scalp electrodes to record the human electroencephalogram (EEG). To date, a large number of studies have analyzed the brain’s higher psychological activities through EEG, which has become an essential tool for analyzing the brain’s senior functions owing to advantages including objectivity, non-invasiveness, and low cost (Cavanagh and Frank, 2014; Anderson and Perone, 2018). With the development of EEG technology, numerous studies explored EEG characteristics at different cognitive levels. Traditionally, EEG is divided according to the frequency bands, namely, the δ rhythm (1–3 Hz), θ rhythm (4–7 Hz), α rhythm (8–13 Hz), β rhythm (14–30 Hz), and γ rhythm (30–80 Hz), which are associated with various aspects of cognitive function (Winneke et al., 2020). Studies have shown that α and θ rhythms are significantly correlated with overall cognitive tests, memory, language, and executive function (Klimesch, 1999). Good cognition and memory performance are significantly correlated with lower theta and higher alpha powers, respectively (Van Der Hiele et al., 2007). The analysis of EEG spectral power in patients with ARHL may be a potential strategy to reveal meaningful cognition-related results. Furthermore, in addition to the EEG power of each frequency band, other characteristics of the frequency bands can also be used as the basis for cognitive evaluation, such as total power, the linear combination of power values in a specific frequency band, average power, and root mean square power (Moretti et al., 2007; Price et al., 2019; Seleznov et al., 2019; Laptinskaya et al., 2020).

**Figure 2:**
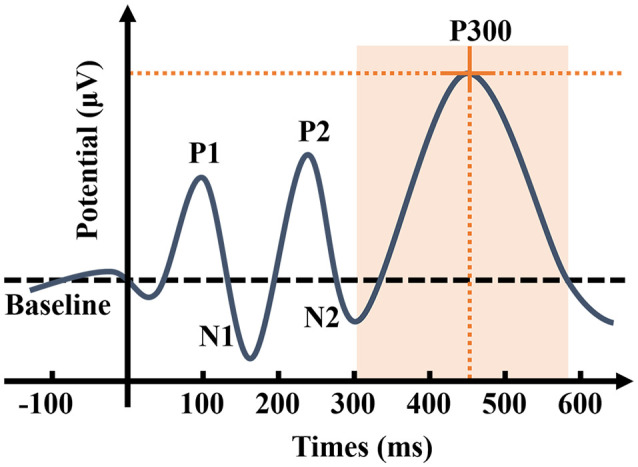
Schematic representation of auditory event-related potential (ERP). P300 is the ERP component triggered by the brain in response to low-probability stimuli, causing a maximum positive wave approximately 300 ms after the stimuli. As the most widely used component in ERP, P300 has two characteristics: amplitude and latency. The P300 amplitude refers to the maximum value that ERP achieves in the time window of 300–500 ms, while peak latency refers to the delay time between the occurrence of the stimuli to the detection of the maximum potential value (Johnson, 1993). P300 amplitude is considered to reflect the attentional resource allocation (Donchin and Coles, 1988; Kok, 2001). P300 latency is considered to reflect the processing speed or efficiency in the process of detecting and evaluating the stimulus (Kutas et al., 1977), and individual differences for P300 latency are correlated with mental function speed, such that shorter latencies are related to superior cognitive performance (Polich, 2007). P300 is affected by numerous factors, such as the concentration of the subject’s attention, the difficulty of the task, the probability of deviation from the stimulus, and the time interval between the two stimuli.

Sutton et al. (1965) proposed event-related potential (ERP). ERPs are closely related to cognitive processes and are therefore regarded as “windows” to “penetrate” mental activities (Helfrich and Knight, 2019). The stimulation path is divided into auditory, visual, and somatosensory ERPs. Auditory ERP components include P1, N1, P2, N2, P300, and mismatch negativity (MMN; Rösler et al., 1986). The P300 is an endogenous ERP component, which is a positive wave that appears about 300 ms after the appearance of the deviant stimuli (Figure 2), mainly related to cognitive activities, such as attention, discrimination, and working memory when people are engaged in a specific task (Polich, 1989; Linden, 2005). The stimulation usually triggers it with the Oddball paradigm (Picton, 1992). In the classic Oddball paradigm, two types of stimuli appear randomly to act on the same sensory channel, and the probability of the stimuli appearing differs considerably (Halgren et al., 1998). Those with high probability, usually 80%, are referred to as standard stimuli, which form the background to the whole experiment, and those with low probability, usually 20%, are called deviant stimuli. The subjects must pay attention to the deviant stimuli and respond as soon as the stimuli appear, which involves pressing a counter or memorizing the number of events of the deviant stimuli, while the standard stimuli remain unnoted. Furthermore, MMN also reflects automatic stimulus discrimination in the human auditory system. Unlike P300, the subjects were able to induce MMN without paying attention to the stimuli. Hence, MMN reflects the activation of an automatic differential detection mechanism in early hearing.

Several EEG studies were carried out on patients with ARHL in recent years. Multiple studies addressing EEG brain functional connectivity and cortical source localization showed that even mild hearing loss could alter functional communication between regions of the cerebral cortex (Bidelman et al., 2019; Price et al., 2019). Long-term hearing loss in patients with ARHL may affect how sound is encoded in the brain, resulting in a cross-modal reorganization of the somatosensory system’s auditory cortex (Cardon and Sharma, 2018; McClannahan et al., 2019). Compared with the elderly with normal-hearing, the temporal lobe cortex activity of patients with ARHL was decreased, whereas frontal cortex activity was increased, which is consistent with the observations in MRI studies (Campbell and Sharma, 2013, 2014; Wingfield and Peelle, 2015). The frontal cortex, which improves sensory perception through top-down regulatory control, is associated with cognitive activities, such as working memory and information processing (Liakakis et al., 2011). This implies that receiving external auditory stimuli triggers changes in the allocation of resources in the cognitive cortex. These changes may be triggered by better compensating for auditory perception, which leads to an increase in the cognitive load, and these results hint at the evidence for the cognitive load hypothesis.

Furthermore, auditory intervention may have the potential to reverse compensatory changes in the cortical resource. One study compared cortical auditory evoked potentials of 32 hearing-impaired seniors aged 62–82 before and after the use of hearing aids (Karawani et al., 2018). The experiment was carried out using the Oddball paradigm, and the results indicated that the amplitude of the P2 component increased significantly after using hearing aids for 6 months. Previous evidence suggested that P2 is a marker of perceptual memory and auditory plasticity (Picton, 2013; Ross et al., 2013). The increase in the P2 amplitude indicates that hearing aids improved hearing ability, working memory performance, and increased cortical neural processing ability. However, this study’s follow-up time was relatively short, and further studies may be necessary to follow patients for a longer time to verify the reliability of the results.

## Discussion

Hearing loss is prevalent in the elderly, and it has been shown that ARHL is independently associated with cognitive decline (Chadha et al., 2017). Determining the mechanism between the two states would be of great theoretical and clinical significance. For individuals with reduced cognitive function, particularly dementia, and Alzheimer’s disease, hearing loss intervention is more comfortable to achieve than for other risk factors (Dawes, 2019). Because pharmacology has not effectively cured hearing impairment, the hearing must be improved by intervention once the diagnosis is confirmed. Currently, hearing aids and cochlear implants are effective methods of hearing loss intervention. Patients with ARHL must promptly conduct hearing compensation or reconstruction to prevent the continued decline of language resolution, which affects interpersonal communication and quality of life. Certainly, auditory interventions significantly improve speech perception and communication skills, affecting social engagement and interaction, and reducing depression, anxiety, and loneliness (Dawes et al., 2015a; Davis et al., 2016). Auditory intervention may be an effective approach to prevent and treat cognitive decline associated with ARHL. The use of an assisted listening device to improve hearing and achieve more participation in social activities may reduce the risk of cognitive decline. Therefore, a comprehensive assessment of cognition over an extended investigation period is highly desirable to understand when and how to intervene with regard to hearing loss and assess the risk of cognitive decline to provide more data and information for clinical diagnosis and treatment.

Although the relationship between ARHL and cognitive decline has yet to be elucidated, we must urgently find a way to identify ARHL patients who are at risk or who are already developing cognitive impairment. Providing audiologists with the opportunity to synchronously test the cognitive status of the elderly during the hearing examination and assess whether it may lead to cognitive impairment would be significant for early detection and prevention. However, a statistical survey of otolaryngologists and audiologists showed that the rate of cognitive function assessment for hearing loss patients was only 21.21% (Raymond et al., 2020), which indicates that the practice of cognitive assessment of high-risk groups, such as patients with ARHL, in clinical practice is not yet universal. This could be because the relationship between the two has not yet been popularized and further due to the lack of objective quantitative indicators with high sensitivity and specificity (Panza et al., 2015; Raymond et al., 2021). The development of electrophysiological technologies, such as EEG and MRI, provides the opportunity to achieve this goal. Current electrophysiological studies reveal a series of structural and functional changes in the brains of patients with ARHL, some of which are not related to age. These changes appear in the auditory and cerebral cortices related to attention and emotional processing (Cardin, 2016; Fitzhugh et al., 2019). In future neuroelectrophysiological and imaging studies, further attention must be paid to patients’ grouping design to confirm the existing findings. Meanwhile, valuable discoveries may be made by analyzing other electrophysiological characteristics.

Currently, the P300 has been widely used to study cognitive function. Studies have shown that with the development of neurodegenerative diseases, the amplitude of P300 decreases while the latency increases (Papadaniil et al., 2016; Tsolaki et al., 2017). Developing studies on auditory ERP in patients with hearing impairment may employ the P300 as an objective examination method to assess patients’ auditory center and cognitive function status with ARHL.

EEG technology has the advantage of high temporal resolution (Michel and Murray, 2012). However, because electrodes measure electrical activity on the brain’s surface, it is difficult to determine whether the signal is generated in the cortex or deeper areas. Hence the spatial resolution is low (Srinivasan, 2006; Michel, 2019). Correspondingly, the spatial resolution of MRI technology is high and can achieve a crisper brain image (Trindade, 2004). However, it takes a few seconds for the blood flow to change during brain activity, and changes in hemodynamic signals in the active brain area detectable by MRI are minuscule. The time interval from when the time point at which the brain is stimulated to the MRI signal’s peak and the limitation of calculation factors during the recording process results in a low temporal resolution (Cheng, 2011). EEG and MRI have the advantages of temporal resolutions down to the ns. scale and spatial resolution down to the micron scale, respectively. However, neither of the technologies can guarantee both good temporal and spatial resolution. Compromises in one dimension are often needed to improve the accuracy in the other.

Ives et al. (1993) achieved the simultaneous acquisition of EEG and MRI for the first time, and presently, simultaneous EEG-fMRI has matured. Simultaneous EEG-fMRI can fuse the time information of the EEG signal’s dynamic changes with spatial information in the large-scale network reflected by fMRI, combining the advantages of fMRI’s high spatial resolution and EEG’s high temporal resolution, making it superior for classifying different cognitive processes (Debener et al., 2006; Abreu et al., 2018). This combination deepens the fusion of multi-modal data, mitigates the shortcomings, overcomes the single-mode method’s limitations, and achieves high-temporal spatial-resolution observation of brain activity, which provides a powerful means for exploring the neural mechanism of psychological nerve activity (Mulert et al., 2008; Ahmad et al., 2016). Its use is expected to be more widespread to formulate more specific and individualized prevention and treatment programs and observe the effects of rehabilitation training for patients with ARHL and cognitive impairment.

### Future Directions

The potential of electrophysiological tools has already been demonstrated in this field, and the following future research directions are preliminarily anticipated:

• Current studies on brain function changes in hearing loss mainly focus on analyzing the resting state of brain function, with few studies addressing the task state. Therefore, researchers are able to design cognitive tasks based on early signs of cognitive declines, such as declines in logic and memory, to simulate daily life scenarios. Hence, dynamic characteristics of the patient’s brain are obtained in the cognitive task state. Here, the cognitive task state refers to how the patients actively perform tasks, such as memory or calculation, rather than passively accepting external stimulation in the form of visual or auditory stimuli. How to ameliorate patient’s participation in the experiment is likewise a relevant challenge when designing experiments.
• Auditory ERP components, such as P300 and MMN, reflect cognitive function, have the advantage of high time resolution, and apply to ARHL patients. By observing the time-domain characteristics of the signal, namely amplitude and latency, the signal reflects the patient’s dynamic cognitive process, which makes it possible to find a potential early biomarker for detection of the process of ARHL patients progressing to cognitive decline.
• As an effective method to study neural activation and endogenous brain activity in the cognitive process, simultaneous EEG-fMRI technology explores the neural mechanism of cognitive activities, which is expected to elucidate the association between ARHL and cognitive decline. Nevertheless, this technology still faces numerous challenges: low signal-to-noise ratio, poor individual comfort, and complex data analysis (Laufs, 2012; Jorge et al., 2014; Murta et al., 2015). Future EEG-fMRI studies still require optimization of algorithms and hardware.
• OAE and ABR also have the potential to determine which ARHL patients could be at risk of having a cognitive impairment (Belkhiria et al., 2019, 2020; Delano et al., 2020). In addition, a recent study found that impaired facial emotion recognition in ARHL patients is correlated with the atrophy of multiple areas of the cerebral cortex and may also relate to cognitive impairment (Belkhiria et al., 2021). Future research may find more reliable biomarkers for cognitive decline caused by ARHL.

EEG and MRI, serving as objective examination tools of advanced brain functions, combined with objective measures of auditory assessment, are expected to evaluate the auditory center and cognitive function status of patients with ARHL. They will, in time, provide a more extensive application for finding clues to search for the causes of ARHL, developing more accurate and individualized prevention and treatment, and observing the effects of rehabilitation training for patients with ARHL and cognitive impairment. Clinical and experimental research results on ARHL patients are abundant; however, many areas remain to be studied. Even if current technical methods have more or fewer limitations, we believe that the joint efforts of experts and researchers globally will eventually reveal the mystery of changes in brain structure and function of hearing loss patients through the use of multi-modal technology, ingenious experimental design, experimental research, sophisticated algorithms, and mature hardware systems. This will enable effective cognitive screening for patients with ARHL, the development of more accurate and valuable treatment methods for ARHL, and their application in clinical practice. The critical link between ARHL and cognitive impairment will be found, leading to combined intervention, and individualized treatment for patients with ARHL will eventually be realized.

## Author Contributions

GN, TY, WW, and YC contributed to the conception and design of the work, and drafting and revising the manuscript for important intellectual content. QZ and ZX drafted corresponding sections of the manuscript. All authors agreed to submit the manuscript in its current state and agree to be accountable for all aspects of the work. All authors contributed to the article and approved the submitted version.

## Conflict of Interest

The authors declare that the research was conducted in the absence of any commercial or financial relationships that could be construed as a potential conflict of interest.
